# Comparative Transcriptomics and RNA-Seq-Based Bulked Segregant Analysis Reveals Genomic Basis Underlying *Cronartium ribicola vcr2* Virulence

**DOI:** 10.3389/fmicb.2021.602812

**Published:** 2021-02-22

**Authors:** Jun-Jun Liu, Richard A. Sniezko, Arezoo Zamany, Holly Williams, Kangakola Omendja, Angelia Kegley, Douglas P. Savin

**Affiliations:** ^1^Canadian Forest Service, Natural Resources Canada, Victoria, BC, Canada; ^2^USDA Forest Service, Dorena Genetic Resource Center, Cottage Grove, OR, United States

**Keywords:** bulked segregant analysis-based RNA-seq (BSR-Seq), candidate effectors, comparative transcriptomics, major gene resistance, white pine-blister rust (WP-BR) interaction

## Abstract

Breeding programs of five-needle pines have documented both major gene resistance (MGR) and quantitative disease resistance (QDR) to *Cronartium ribicola* (Cri), a non-native, invasive fungal pathogen causing white pine blister rust (WPBR). WPBR is one of the most deadly forest diseases in North America. However, Cri virulent pathotypes have evolved and can successfully infect and kill trees carrying resistance (*R*) genes, including *vcr2* that overcomes MGR conferred by the western white pine (WWP, *Pinus monticola*) R gene (*Cr2*). In the absence of a reference genome, the present study generated a *vcr2* reference transcriptome, consisting of about 20,000 transcripts with 1,014 being predicted to encode secreted proteins (SPs). Comparative profiling of transcriptomes and secretomes revealed *vcr2* was significantly enriched for several gene ontology (GO) terms relating to oxidation-reduction processes and detoxification, suggesting that multiple molecular mechanisms contribute to pathogenicity of the *vcr2* pathotype for its overcoming *Cr2*. RNA-seq-based bulked segregant analysis (BSR-Seq) revealed genome-wide DNA variations, including about 65,617 single nucleotide polymorphism (SNP) loci in 7,749 polymorphic genes shared by *vcr2* and avirulent (*Avcr2*) pathotypes. An examination of the distribution of minor allele frequency (MAF) uncovered a high level of genomic divergence between *vcr2* and *Avcr2* pathotypes. By integration of extreme-phenotypic genome-wide association (XP-GWAS) analysis and allele frequency directional difference (AFDD) mapping, we identified a set of *vcr2*-associated SNPs within functional genes, involved in fungal virulence and other molecular functions. These included six SPs that were top candidate effectors with putative activities of reticuline oxidase, proteins with common in several fungal extracellular membrane (CFEM) domain or ferritin-like domain, polysaccharide lyase, rds1p-like stress responsive protein, and two Cri-specific proteins without annotation. Candidate effectors and *vcr2*-associated genes provide valuable resources for further deciphering molecular mechanisms of virulence and pathogenicity by functional analysis and the subsequent development of diagnostic tools for monitoring the virulence landscape in the WPBR pathosystems.

## Introduction

White pine blister rust (WPBR) caused by *Cronartium ribicola* (*Cri*) J.C. Fisch is the most severe and damaging disease of five-needle pine species (subgenus *Strobus*) world-wide. This fungal pathogen depends on pine trees as its primary hosts and Ribes as the principal alternate hosts to complete its life-cycle (Geils et al., 2010). Following its accidental introduction to North America in 1906, Cri had spread to most geographical regions where commercial five-needle pines were originally distributed by the 1950s (Maloy, 2018). All nine native five-needle pine species in the United States, including four native to Canada, are very susceptible to WPBR (Tomback and Achuff, 2010).

*Cronartium ribicola* has a complex life cycle with five spore stages (aeciospores, urediniospores, teliospores, basidiospores, and spermatia), requiring an alternate hosts (mainly species of Ribes in North America, and Pedicularis in some areas of Asia) to complete its life cycle over at least 2 years. Of the five spore stages of the Cri life cycle, only basidiospores are able to infect pine needles, which occurs in late summer or early fall when they develop on the underside of infected Ribes leaves during cool, wet weather conditions. Basidiospores germinate on the needle surface, and their germ tubes penetrate into needle tissues through stomatal pores. The mycelia grow at infection sites and spread to stems through vascular tissues, and stem cankers develop in the coming spring of next year. As an exotic, invasive fungal pathogen, Cri directly kills western white pine (WWP; *Pinus monticola* Dougl. ex D. Don) and other native five-needle pines of all ages across North America and predisposes older trees to secondary attack by insects and other fungi.

Due to the high economic and ecological importance of five-needle pines, screening of host resistance to this rust fungus to develop resistant populations is seen as the key to using these species in restoration and reforestation. In some species this work has been ongoing for more than 50 years, and different levels and types of resistance have been found among five-needle pine species (Sniezko et al., 2014, 2020). Resistance (*R*) loci for major gene resistance (MGR) have been found in several five-needle pine species. Host trees with different resistant genotypes have allowed distinct virulent pathotypes to evolve from predominantly avirulent wild-type races, overcoming MGR in sugar pine and WWP (Kinloch et al., 2004), as well as in Ribes (Tanguay et al., 2013). The Cri pathotype *vcr2* was discovered because of its ability to kill WWP trees with the major R gene (*Cr2*). *vcr2* was originally detected in the Champion Mine population on the Umpqua National Forest in Oregon, a presence probably as early as the mid-1950s (Kinloch et al., 2004); and it has now been documented in much of western Oregon and at Happy Camp, CA, United States (Kinloch et al., 2003). *Cr2* progeny in field trials in Washington now show infection, suggesting that *vcr2* may arise, or migrate from western Oregon. These observations suggest that the Cri virulent pathotype arises relatively quickly under selection where *Cr2* trees are present. Planting resistant seedlings is seen as the most effective and environmentally friendly way to manage WPBR. However, increased field planting of resistant trees also promotes selection pressure on Cri and accelerates the spread of *vcr* races and other genotypes with enhanced virulence. More information is required about compatible and incompatible white pine-blister rust (WP-BR) interactions to understand the durability and stability of MGR and quantitative disease resistance in different species (Sniezko et al., 2020).

Understanding the mechanisms underlying variations in pathogenicity is necessary for disease management and utilization of tools for genetic resistance. However, the pathogen side of molecular tree-microbe interactions is poorly understood in WPBR pathosystems. Previously, investigation of Cri genetic diversity revealed the global movement, local host selection, and genetic shifts in local populations (Richardson et al., 2008; Brar et al., 2015). Despite this progress, the absence of a Cri reference genome sequence means that little molecular information is available for the *Cronartium* genus. Recently, the genome of *Cronartium quercuum* f.sp. fusiforme (Cqf) was sequenced (Pendleton et al., 2014), allowing comparative genomics studies to identify avirulence genes and secreted effectors in this rust genus. Transcriptome profiling of Cri avirulent pathotypes described genes and putative effectors that were differentially expressed among different stages of its life cycle (Liu et al., 2015). An effector secreted by Cri avirulent pathotypes was functionally characterized (Ma et al., 2019). Such genomics and transcriptomics studies are invaluable in the discovery of gene functions and metabolic pathways in this rust group. However, genetics and molecular understanding of the genomic basis of Cri virulence is still lacking at this point (Kinloch et al., 2004).

In this paper, we report on the profiling of Cri *vcr2* transcriptomes and secretomes. To understand the molecular mechanisms contributing to pathogenic virulence, pathotype-specific and conserved transcriptomic programs were revealed by a comparative transcriptomic approach. Candidate effectors and *vcr2*-associated genes were further identified by RNA-seq-based bulked segregant analysis (BSR-Seq). These genomic resources provide a strong foundation for future efforts to isolate the *vcr2* and *Avcr2* genes for investigations of their biological properties and development of new diagnostic tests for monitoring virulence across Cri’s landscape.

## Materials and Methods

### Fungal and Plant Materials

*Cronartium ribicola* (Cri) *vcr2* aeciospores were collected from infected trees of wind-pollinated progeny of two Cr2/Cr2 parent trees, 20046-031 and 023225 on March 12, 2014. Their seedlings were planted as part of a field trial (RV7) in 2000 at Travis Tyrrell seed orchard, Oregon, United States (Sniezko et al., 2020), where *vcr2* was documented (Kinloch et al. 2004). All Cri aeciospore samples were collected separately from different trees as biological repeats. Infected stems showing disease symptoms of discolored bark tissues were collected from 20-month-old seedlings ~14 months post Cri infection as described previously (Liu et al., 2014). In brief, seeds were sown in June 2010 after 4 months stratification. Seedlings were grown in a greenhouse and inoculated with Cri basidiospores in September 2010 using infected leaves of Ribes spp. (the alternate host of *C. ribicola*). The Cri sources in these inoculations were collected from eight field locations in eastern Oregon as a heterogenous mixture. Phenotypic traits were assessed at periodic intervals in 2011 when infection symptoms were evident on needles and stems. Based on phenotypes of needle disease spot types (i.e., all HR-like; all susceptible; mixed; and un-identified disease spots) and stem symptoms (i.e., cankers present or absent), each seedling was determined as a resistant (*Cr2*/−) or susceptible (*cr2/cr2*) genotype, and their genotypes were further confirmed by SNP genotyping using *Cr2*-linked DNA markers (Liu et al., 2020). Cankered WWP (*Cr2*/−) stems were harvested for the *vcr2* pathotype with three biological repeats, each pooled with at least 15 seedlings. Uninfected healthy seedlings were used as negative controls. The samples were frozen using liquid nitrogen and stored at −80°C before RNA extraction.

### RNA-Seq Analysis and *de novo* Assembly and Post Filtering of the Transcriptomes

Total RNAs were extracted from aeciospores and cankered WWP stems following a protocol described previously (Liu, 2012). After genomic DNA digestion with DNase, RNA was re-purified using an RNeasy plant mini kit (Qiagen). Following mRNA separation using an RNA-seq sample preparation kit (Illumina, San Diego, CA, United States), cDNA libraries were constructed with sample-specific 6-bp bar-coding tags. Paired-ends (PE) were sequenced on an Illumina HiSeq 2500 instrument (Illumina), which yielded 23–56 million 100-bp PE reads per library at the National Research Council of Canada (Saskatoon, Canada). Raw reads of the Illumina RNA-seq 100-bp PE sequences from nine samples were deposited in the NCBI SRA under accession number SRR11101722–SRR11101730.

Trimmomatic with default settings was used to remove adapter sequences and low-quality bases (phred score <20) from the raw RNA-seq reads (Bolger et al., 2014). The clean reads were *de novo* assembled into transcripts for the aeciospores and cankered stems separately using Trinity (version: trinityrnaseq_r2013-02-25) with default k-mer length of 25 (Haas et al., 2013). A workflow as outlined by De Fine Licht et al. (2017) was used to remove host transcripts and reduce sequence redundancy. Putative WWP transcripts were first removed from two assemblies by BLASTn search (Altschul et al., 1990) against healthy WWP stem transcriptome (Liu et al., 2014) with a cut-off at e-value < e-6. The highest expressed isoform was then selected for each component using “pick_isoform_trinity_RSEM.py” (Yang and Smith, 2013), and overlapping sequences were assembled into contigs using CAP3 Sequence Assembly Program with setting -o 200 -p 99 (Huang and Madan, 1999). After combining contigs and singlets, CD-Hit-EST (Fu et al., 2012) was run to combine transcripts at 97% identity of the nucleotide sequences. Finally, each sequence was assigned to a putative taxonomical origin with BLASTx searches (e-value < 1e-6) against the UNIPROT database (ver. 2015_1) using Metagenome Analyzer (MEGAN, ver 5.7.1; Huson et al., 2011). Transcripts were assigned to four categories: “fungi”, “no hits” (sequences for which BLASTx did not find any hits), “not assigned” (sequences had BLASTx hits with bitscores below the threshold, thus no taxonomy was assigned to them), and “other” for those sequences that were assigned a taxonomy but beyond any of the above three categories. Sequences in the category of “not assigned” were also excluded where BLASTx search against the proteome of *C. quercuum* f. sp. fusiforme (Pendleton et al., 2014; downloaded at https://mycocosm.jgi.doe.gov/Croqu1/Croqu1.home.html) had E values >e-6. The sequences were further filtered by total length >200-bp with a minimum open reading frame (ORF) long 50 codons as predicted by TransDecoder. The putative protein sequences were combined by a run of CD-Hit at 97% of amino acid sequence identity. The resulting transcript dataset, including all sequences in the categories “fungi” and “no hits” and a part of “not assigned” sequences, was considered as a *vcr2* reference transcriptome for further analysis. This transcriptome shotgun assembly project has been deposited at DDBJ/EMBL/GenBank under the accession GIKE00000000 in Bioproject PRJNA261951.

Ortholog cluster analysis was performed to compare the reference transcriptomes between *vcr2* and *Avcr2* (Liu et al., 2015) using OrthoVenn2 with the default settings of E-values ≤1e-5 and an inflation value of 1.5 (Xu et al., 2019). Gene names and gene ontology (GO) terms were assigned to the Cri genes based on their homologies to the available databases (NCBI-nr, PIR, KEGG, and GO) using BLAST2GO (Götz et al., 2008). GO-term enrichment was analyzed by Fisher’s exact test with correction for multiple testing (*p* values < 0.01) as implemented using BLAST2GO.

### Prediction of Secreted Proteins

The *vcr2* secretome was determined in complete ORFs based on bioinformatic prediction. The N-terminal signal peptide was predicted by combining SignalP v5.0 (Petersen et al., 2011) and Phobius (Kall et al., 2004). Transmembrane (TM) domains were predicted by allowing one TM domain in the first 60 amino acids using TMHMM^1^; and proteins with endoplasmic reticulum (ER) targeting sequence (Prosite: PS00014) were removed by using ScanProsite (de Castro et al., 2006). CD-HIT was used to cluster secreted proteins from *vcr2* and *Avcr2* (Liu et al., 2015) into clusters at a threshold of 98% amino acid sequence identity. Localization of effectors to apoplast was predicted by ApoplastP based on depletion in glutamic acid, acidic amino acids and charged amino acids and enrichment in small amino acids (Sperschneider et al., 2018). Carbohydrate-active enzymes (CAZy) in the secretome were annotated using the dbCAN2 meta server (Zhang et al., 2018). Pfam domain predictions were performed using HMMSCAN at the European Bioinformatics Institute (EMBL-EBI).^2^

### Global Gene Expression Analysis

For comparative transcriptomic study of *vcr2* with *Avcr2*, RNA-seq 100-bp PE reads from *Avcr2*-related samples were downloaded from the NCBI SRA. They included cankered stems of susceptible (*cr2/cr2*) WWP seedlings infected by the *Avcr2* pathotype (SRR3273235–SRR3273237), *Avcr2* aeciospores (SRR1583540, SRR1583545, and SRR1583552), avcr2 urediniospores (SRR1583557–SRR1583559), as well as the shoot-tip samples of healthy WWP (*Cr2*/−) seedlings (SRR1574690–1574692) as negative controls.

RNA-Seq reads were mapped back to the *vcr2* transcriptome using CLC with parameters as: no masking, mismatch cost = 2, insertion/deletion cost = 3, length fraction = 0.9, similarity fraction = 0.9, auto-detect paired distances = yes, global alignment = yes, and non-specific match handling = ignore. As only paired reads were counted, transcript expression values were normalized by fragments per kilobase of exon per million fragments mapped (FPKM, Mortazavi et al., 2008) using CLC Genomics Workbench 5.5 (CLC bio, QIAgen, Aarhus, Denmark). Genes were counted as expressed in a specific pathotype using a cut-off of an average FPKM ≥ 1 in all sample types. Differentially expressed genes (DEGs) were identified by Baggerley’s test with weighted proportions fold change > |2| and Bonferroni corrected *p* < 0.05.

### Identification of DNA Polymorphisms and Estimation of Minor Allele Frequency

Using the *vcr2* reference transcriptome *de novo* assembled in this study as a reference, the variant detection tool embedded in CLC Genomics Workbench was used to analyze DNA variants in *vcr2* and *Avcr2* pathotypes as two pooled samples. Clean RNA-seq reads were pooled from six and 12 samples for the *vcr2* and *Avcr2* pathotypes separately. The two sets of pooled reads were mapped to the reference using CLC with parameters set the same as in the above gene expression study. DNA variants were called by the quality-based variant detection method using CLC with parameters set as: neighborhood radius = 5, maximum gap and mismatch count = 2, minimum neighborhood quality = 15, minimum central quality = 20, ignore non-specific matches = yes, ignore broken pairs = no, minimum coverage = 4, minimum variant frequency (%) = 1.0, and maximum expected alleles = 2. DNA variants were further filtered by a minimum contig depth of 10 reads covering the polymorphic site. These genes with low expression levels were not included in further analysis of genetic diversity. The *vcr2* and *Avcr2* pools were compared to identify variants specific to either pool as well as those common to both pools. Minor allele frequency (MAF) was estimated in the two data sets based on pathotypes. The distribution of MAFs was estimated according to the proportion of SNPs with MAF values that fall within the following six ranges: < 0.05, ≥0.05 to < 0.1, ≥ 0.1 to < 0.2, ≥ 0.2 to < 0.3, ≥ 0.3 to < 0.4 and ≥ 0.4 to ≤ 0.5. Chi-squared tests were used to compare frequency differences between the two pathotypes.

### Detection of *vcr2*-Associaed SNPs

Bulked segregant analysis RNA-seq (BSR-Seq, Michelmore et al., 1991; Liu et al., 2012; Trick et al., 2012) was used to detect *vcr2*-associated SNPs. RNA-seq reads were bulked from individual samples from at least 90 *C ribicola* infected WWP trees based on *vcr2* and *Avcr2* pathotypes and mapped to the reference transcriptome as described above to calculate sequencing depth of the SNPs, counts of reference and alternative alleles at each SNP locus, as well as allele frequency for each SNP across the transcriptome throughout the whole genome.

DNA variants common to both *vcr2* and *Avcr2* pools were used to detect *vcr2*-associated SNPs, which are genetically informative due to the elimination of false positive variants, a process which is challenging in NGS data processing (Ribeiro et al., 2015). Extreme-phenotype genome-wide association study (XP-GWAS) and allele frequency directional difference (AFDD) mapping were integrated to identify *vcr2*-associated SNPs.

Extreme-phenotype genome-wide association study was generally performed using a generalized linear mode as described by Yang et al. (2015). To attenuate over-dispersion of the *X*^2^ test statistic from RNA-seq data with large variation of gene expression levels, counts of reference and alternative alleles were first normalized based on allele frequency for each SNP loci prior to the XP-GWAS run, where normalized count = log_10_ (count/10) x the allele frequency in percentage point (i.e., between 1 and 100). Phenotypic pool numbers were set as 1 = vcr1, 3 = Avcr2, and 2 = random that was generated by randomly pooling RNA-seq reads equally from both *vcr2* and *Avcr2*. The likelihood ratio test statistic was computed for testing the null model of no association between success probability and phenotypic pool number; and then corrected by dividing by *λ*, an inflation factor proposed for consideration of cryptic within-group relatedness (Devlin and Roeder, 1999). The inflation factor (λ) was estimated using the R add-on package “gap”.^3^ To identify significant variants, the values of *p* derived from the likelihood ratio test statistic were corrected by the FDR method at the 5% level (Yang et al., 2015).

Allele frequency directional difference mapping was performed using two pooled samples as described previously (Fekih et al., 2013; Takagi et al., 2013). *Avcr2* was presumed to be a dominant trait while virulent races, expected to be homozygous (*vcr2/vcr2*), were relatively rare across Cri landscape. Based on this hypothesis, genotypes were proposed for the bulked samples of virulence (**ll**) and avirulence (**l**m), where the allele “**l**” is assumed to be linked to *vcr2*. Under this scenario, the expected frequencies of the *vcr2*-linked alleles were close to 100% in the *vcr2* pool and close to 50% in the *Avcr2* pool. Therefore, SNP loci with allele frequency >0.90 as homozygote (**ll**) in the *vcr2* pool and allele frequency in the range of 0.4~0.6 as heterozygote (**l**m) in the *Avcr2* pool were selected for association testing.

Although considered less likely, the opposite hypothesis was also considered where the *vcr2* pathotype has a heterozygous genotype (**l**m) and the *Avcr2* pathotype has a homozygous genotype (mm). Under this assumption, the *vcr2*-linked allele (**l**) would have frequencies close to 50% in the *vcr2* pool and its frequency would be close to 0 in the *Avcr2* pool. SNP loci were then selected for virulence association tests with allele frequency in the range of 0.4~0.6 as heterozygote (lm) in the *vcr2* pool and allele frequency < 0.1 as the homozygote (mm) in the *Avcr2* pool. Under both assumptions, the AFDD threshold was set with the minimum of 30% points with the goal being to search for variants with AFDD of 50% points as a practical measurement (Fekih et al., 2013; Dougherty et al., 2018). The AFDD values were plotted against values of *p* from XP-GWAS for identification of significant association.

## Results

### *De novo* Assembly of the Cri *vcr2* Reference Transcriptome by RNA-Seq Analysis

A total of 102 and 161 million 100-bp RNA-seq PE reads were generated from RNA prepared from *vcr2* aeciospores and cankered WWP stems with *Cr2*/− genotypes, respectively, (Supplementary Table S1). Because infected host tissues contained a mixture of mRNAs expressed by both *C. ribicola* mycelia and WWP cells, transcriptomes were *de-novo* assembled for the spore samples and cankered WWP stem samples separately. The Cri samples collected in the present study came from greenhouses and the field, because culturing rust fungi *in vitro* is very difficult. In addition to Cri and WWP, a wide range of other organisms might contribute to RNA-seq reads of these environmental samples. After removal of host WWP transcripts, MEGAN analysis assigned transcripts and RNA-seq reads into the four categories: fungi, no hits, not assigned, and other. Although fungal transcripts accounted for only 54.2% of total *de novo* assembled transcriptomes, 93.3% of total mapped RNA-seq reads were assigned to them (Supplementary Figure S1). Detailed analysis of the WWP host transcriptome and biodiversity of the WPBR field samples will be described in a separate report. Following removal of transcripts potentially expressed by other taxa from the primary assemblies by bioinformatic processing as outlined in the methods, a final set of 24K unique transcripts was generated, representing the Cri *vcr2* reference transcriptome with a total length of ~22 Mb (GenBank TSA accession GIKE00000000) for further analysis. Searching open reading frames (ORFs) at a cut-off of 150-bp identified 6,859 transcripts with putatively complete ORFs, while the remainder had incomplete ORFs, with a total ORF length of ~15 Mb (Supplementary Table S2).

This *vcr2* reference transcriptome was compared to that of the Cri *Avcr2* pathotype (Liu et al., 2015) using OrthoVenn2 (Supplementary Table S3). Both pathotypes had a similar number of clusters (8,498 vs. 8,063), but singleton clusters (proteins that do not cluster with others) were about two times more prevalent in *vcr2* than in *Avcr2* (15,793 vs. 6,324). The Cri *vcr2* and *Avcr2* pathotypes shared 6,944 clusters between them while 435 and 1,119 unique clusters were detected in *vcr2* and *Avcr2*, respectively (Supplementary Figure S2). Both the unique clusters and the unique singletons might represent novel pathotype-specific proteins, or they may reflect differences in filtering of the transcriptome assemblies. OrthoVenn2 analysis did reveal that 435 *vcr2*-unique clusters were enriched with three GO terms of biological processes (cellulose catabolic process, hydrogen peroxide catabolic process, and mitochondrial electron transport, cytochrome *c* to oxygen) while 1,119 *Avcr2*-unique clusters were enriched with only one biological process termed as translation (Supplementary Table S4).

### Prediction of the Cri Secretome

**Figure 1:**
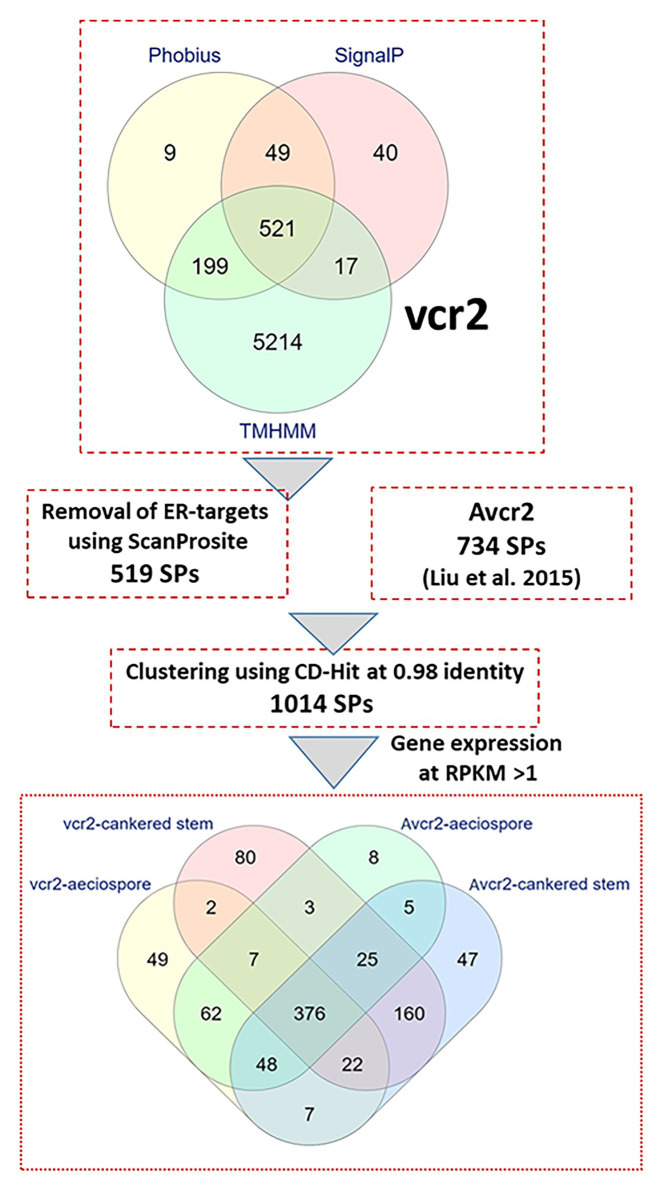
In-silico secretome prediction and identification of pathotype-specific effectors using a combination of computational programs. Putative full-length proteins were used to predict presence of signal peptides by using SignalP-v5.0 and Phobius. Proteins with transmembrane (TM) domains were removed by using TMHMM-v2.0 and Phobius. Proteins targeted to endoplasmic reticulum (ER) were removed by using ScanProsite. Cri secreted proteins from *vcr2* and *Avcr2* pathotypes were clustered by using CD-Hit. Within the Cri secretome, pathotype-specific effectors and those exclusively expressed in cankered host tissues were determined by mapping RNA-seq reads with a threshold of FPKM >1. The Venn diagram at bottom showing number of unique and shared proteins identified in Cri *vcr2* and *Avcr2* secretomes.

Fungal pathogens secrete effector proteins into the plant apoplast or cytoplasm to trigger molecular plant-microbe interactions. The full-length proteins encoded by the 6,859 complete ORFs were used to mine secreted proteins (SPs) in the Cri *vcr2* reference transcriptome. Presence of secretory signal peptides at the N termini of proteins and absence of transmembrane domains were used to identify candidate effectors. A combination of four in-silico prediction programs (SignalP-v5.0, Phobius, TMHMM, and ScanProsite) revealed 519 candidate genes expressed as secreted proteins in the Cri vcr2 reference transcriptome (Figure 1), which accounted for 7.6% of unigenes with complete ORFs. After clustering with a set of 734 proteins identified previously as the Cri avcr secretome (Liu et al., 2015) and by using CD-Hit with a threshold of 98% identity between amino acid sequences, a total of 1,014 SPs were identified in transcriptomes of both *vcr2* and *Avcr2* pathotypes (Figure 1; Supplementary Table S5).

B2G based annotation showed 687 SPs had significant hits (Blastx *E* values < e-6) in the GeneBank nr database. Of all SPs, 109 and 321 SPs were further classified within CAZy and apoplast groups, respectively, (Supplementary Table S6). In addition, HMMScan revealed Pfam domains in 360 Cri SPs (Supplementary Table S6). The secreted CAZyes included 63 glycoside hydrolases (GHs), 3 glycosyltransferases (GTs), 4 polysaccharide lyases (PLs), 20 carbohydrate esterases (CEs), and 19 proteins with redox-active auxiliary activities (AAs). Protease/peptidase-like proteins comprised 29 annotated SPs in the Cri secretome, while others included multiple members of gene families for multi-copper oxidase laccase-like proteins, endoglucanases, nucleases, peroxidases, L-ascorbate oxidases, thioredoxins, Cu/Zn superoxide dismutase (SOD), pathogenesis-related proteins (PR-1-like and thaumatin-like proteins), catalases, and expansin proteins, Egh16-like virulence factors, common in several fungal extracellular membrane proteins (CFEM)-domain proteins, heat shock proteins (HSPs) and their chaperones with DnaJ domain (Supplementary Table S6). Hypothetical proteins with unknown function comprised 53.6% of the total SPs, but the majority had top Blast hits related to species of other rust fungi belonging to the genus *Melampsora* and *Puccinia* (Supplementary Table S6).

### Expression Profiling of the Transcriptomes Revealed Pathotype-Specific Pathways

**Figure 2:**
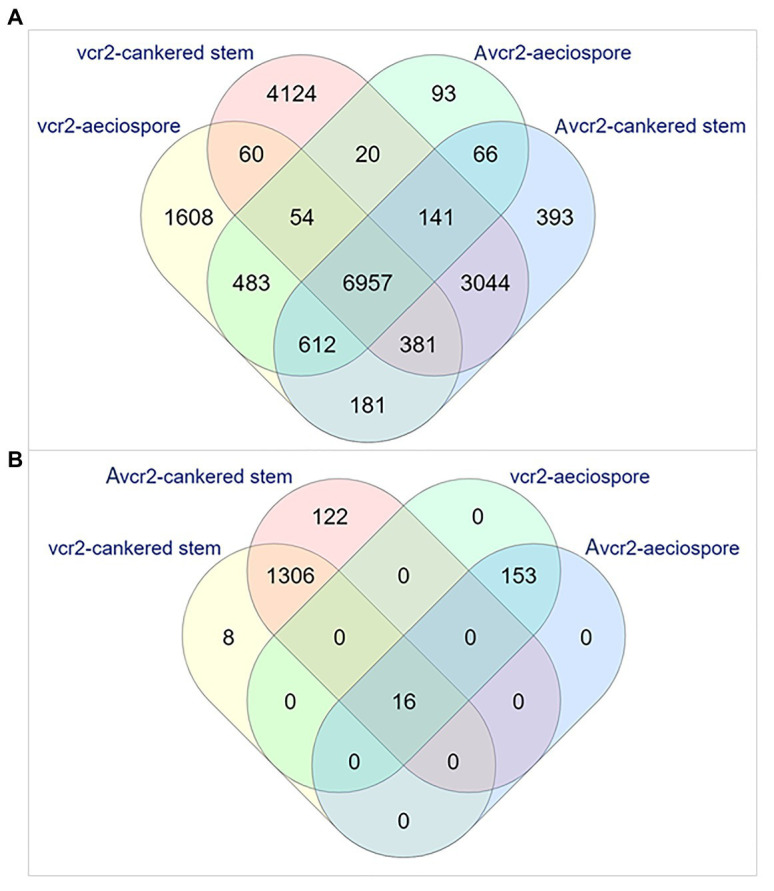
Venn diagrams representing numbers of the expressed unigenes. The expressed unigenes were counted in aeciospore and cankered stem with FPKM > 1. **(A)** Overlap of total genes expressed in *vcr2* and *Avcr2* pathotypes. **(B)** Distribution of differentially expressed genes (DEGs) by comparison of *vcr2* with *Avcr2*.

Expression profiling was performed by mapping RNA-seq reads to the Cri *vcr2* reference transcriptome, including the Cri secretome. About 72~73% of total RNA-seq reads from aeciospores were mapped, while mapped reads from cankered WWP stems only accounted for 0.08~8% of the total RNA-seq reads (Supplementary Table S1). Abundance of fungal RNA-seq reads was much lower in *vcr2*-infected stems of the WWP resistant trees with *Cr2*/− genotype than in *Avcr2*-infected stems of the WWP susceptible trees with *cr2/cr2* genotype (0.5 ± 0.6% vs. 7.6 ± 0.9%, *t*-test *p* = 1e-3). Transcripts expressed in pathotypes were filtered by a cut-off of FPKM = 1 on average, per sample type, resulting in detection of 18,014 and 12,771 genes expressed in *vcr2* and *Avcr2* pathotypes, respectively, (Supplementary Table S1). A core set of 7,092 genes was expressed in both *vcr2* and *Avcr2* pathotypes for both aeciospores and infected WWP stems (Figure 2A).

**Figure 3:**
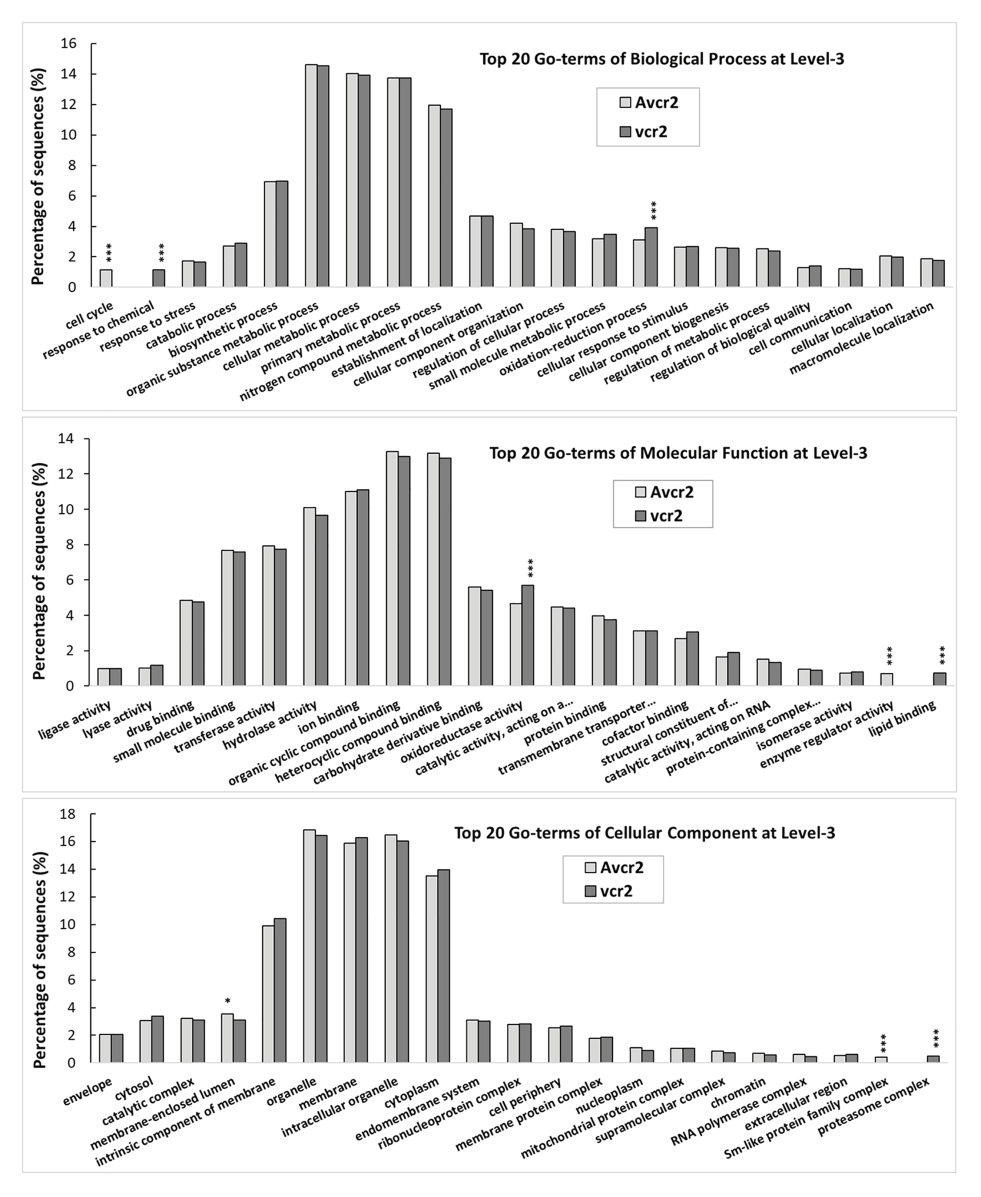
GO-term comparison between *vcr2* and *Avcr2* transcriptomes. The *vcr2* and *Avcr2* transcriptomes consist of 18,596 and 13,139 transcripts with the mean FPKM >1. A comparison of the top 20 GO-terms at level-3 shows the *vcr2* transcriptome with a significant enrichment of oxidation-reduction process (GO:0055114), response to chemical (GO:0042221), oxidoreductase activity (GO:0016491), lipid binding (GO:0008289), and proteasome complex (GO:0000502) while the *Avcr2* transcriptome with enrichment of cell cycle (GO:0007049), enzyme regulator activity (GO:0030234), membrane-enclosed lumen (GO:0031974), Sm-like protein family complex (GO:0120114). One and three stars indicate chi-square test *p* < 0.05 and 0.001, respectively.

Following B2G-based gene annotation, a comparison of the top 20 GO-terms at level-3 shows both pathotypes shared five main biological processes (biosynthetic process, cellular metabolic process, organic substance metabolic process, primary metabolic process, and nitrogen compound metabolic process), each accounting for >10% of the total annotated transcripts. Consistently with results of orthologous clustering analysis by OrthoVenn2, *X*^2^ test revealed that the *vcr2* transcriptome had significant enrichment of oxidation-reduction process (GO: 0055114, *p* < 1e-5), response to chemical (GO: 0042221, *p* < 1e-5), oxidoreductase activity (GO: 0016491, *p* < 1e-3), lipid binding (GO: 0008289, *p* < 1e-5), and proteasome complex (GO: 0000502, *p* < 1e-5) while the *Avcr2* transcriptome had enrichment of cell cycle (GO: 0007049, *p* < 1e-5), enzyme regulator activity (GO: 0030234, *p* < 1e-5), membrane-enclosed lumen (GO: 0031974, *p* < 3e-2), and Sm-like protein family complex (GO: 0120114, *p* < 1e-5; Figure 3).

Examination of pathotype-specific transcripts detected 1,610 and 4,178 genes exclusively expressed in *Avcr2* aeciospores and cankered stems of WWP seedlings with genotypes *Cr2*/–, respectively. Relatively fewer genes were detected with *Avcr2*-specific expression patterns with 99 and 437 genes in aeciospores and cankered WWP stems, respectively, (Figure 2A). Similar to the transcriptome results shown above, Fisher’s exact tests showed *vcr2*-specifically expressed genes significantly enriched with seven GO-terms of molecular functions and 16 GO-terms of biological processes (FDR-adjusted *p* < 1e-3; Supplementary Table S7). The most enriched biological processes included oxidation-reduction process (GO: 0055114), carbohydrate metabolic process (GO: 0005975), detoxification (GO: 0098754), cellular response to toxic substance (GO: 0097237), involving 568, 230, 74, and 73 genes, respectively. Consistently, the most enriched molecular functions included oxidoreductase activity (GO: 0016491), cofactor binding (GO: 0048037), and coenzyme binding (GO: 0050662). In contrast, there were no enriched GO term detected in *Avcr2*-specific transcripts.

In addition to the exclusive presence or absence of transcripts, a total of 1,605 DEGs (Bonferroni adjusted *p* < 0.05 and fold changes ≥ 2) were detected between *vcr2* and *Avcr2* pathotypes, including 169 in aeciospores and 1,452 in cankered stems (Figure 2B). Of these, 8 and 122 DEGs were not detectable (FPKM = 0) in cankered stems infected by *Avcr2* and *vcr2*, respectively (Figure 2B). The majority of aeciospore DEGs (160 of 169) were upregulated in *vcr2* compared to *Avcr2*, while the majority of cankered stem DEGs (1,361 of 1,452) were upregulated in *Avcr2* compared to *vcr2*. GO enrichment revealed seven biological processes enriched in DEGs in aeciospore (FDR-adjusted *p* < 1e-3), including cellular process, macromolecule modification, regulation of cellular component organization, cellular component organization or biogenesis, cellular protein modification process, regulation of organelle organization, and organelle organization. DEGs in cankered stems were enriched with four other biological processes (FDR-adjusted 0.01 < *p* < 0.05), nucleic acid metabolic process, macromolecule modification, regulation of nucleic acid-templated transcription, and regulation of cellular process (Supplementary Table S7).

### Expression Profiling of the Cri secretome Identified *vcr2* Candidates

Most SPs belonged to gene families with multiple members with differential expression among family members. Among the top 60 genes expressed at the highest levels (FPKM > 1,000) in infected pine stems, 40 of them were predicted to be SPs and annotated with functions such as GH5, GH12, GH17, GH18, GH27, PL3, HSP12, subtilisin protease, and thaumatin-like protein (Supplementary Table S8). A total of 901 SPs were found with average FPKM >1; and 376 of those were expressed in both pathotypes in aeciospores and mycelium growing inside the pine stems (Figure 1). These conserved genes likely represent a core group of effector candidates enriched for effectors that may be essential for successful fungal infection into five-needle pines and Ribes.

Furthermore, 131 and 60 SPs were specifically expressed in either the *vcr2* or *Avcr2* pathotype, respectively, at a cut-off of FPKM ≥ 1 (Figure 1; Supplementary Table S6). Of these, 36 and 16 SPs were not detectable in the other pathotype, i.e., with FFPKM = 0 in any of the *Avcr2* and *vcr2* samples. Specifically expressed SPs in *vcr2* cankered stems were annotated as ATP-NAD kinase, cutinase 1, endo-beta-1,6-glucanase, exonuclease, GT24, CE8, isoleucyl-tRNA synthetase, ligase, N-acylphosphatidylethanolamine-specific phospholipase D (NAPEPLD), nucleotidyltransferase, oxidoreductase, PR-1-like protein, protein disulfide isomerase (PDI), protein disulfide oxidoreductase (PDOR), subtilisin-like protein, UDP-N-acetylglucosamine-dolichyl-phosphate N-acetylglucosaminephosphotransferase, and other hypothetical proteins. In contrast, few specifically expressed SPs in *Avcr2* cankered stems were annotated; this list included homologs to ATP-NAD kinase, carboxylesterase, PL1, GH5, GH8, and pupal cuticle 27-like protein. Comparisons between expression profiles and functional annotations revealed that the SPs specifically expressed in *vcr2* cankered stems most likely have an effect on the virulence of *vcr2* pathotype for overcoming *Cr2*-conferred resistance.

In addition, 131 of the 1,605 DEGs identified above, were predicted to be SP-coding DEGs, of which 93% (122/131) were candidate effector genes upregulated in *Avcr2* cankered stem compared with *vcr2*. B2G analysis revealed that 77% of the DEGs (101/131) had significant homology hits to CE4, CBM13 and 18, GH of various families, PL3, proteases/peptidase, peroxidase, thaumatin-like protein, aromatic compound dioxygenases, and others. Apparently, the majority of these differentially expressed SPs are conserved and play indispensable roles during tree infection in compatible *Cr2-Avcr2* interactions. In contrast, only four SP-coding DEGs were upregulated in *vcr2* cankered stem, annotated as cutinase, GH10, GH61 domain protein, and hypothetical protein.

### Genomic Variations and Association With Cri Virulence

**Figure 4:**
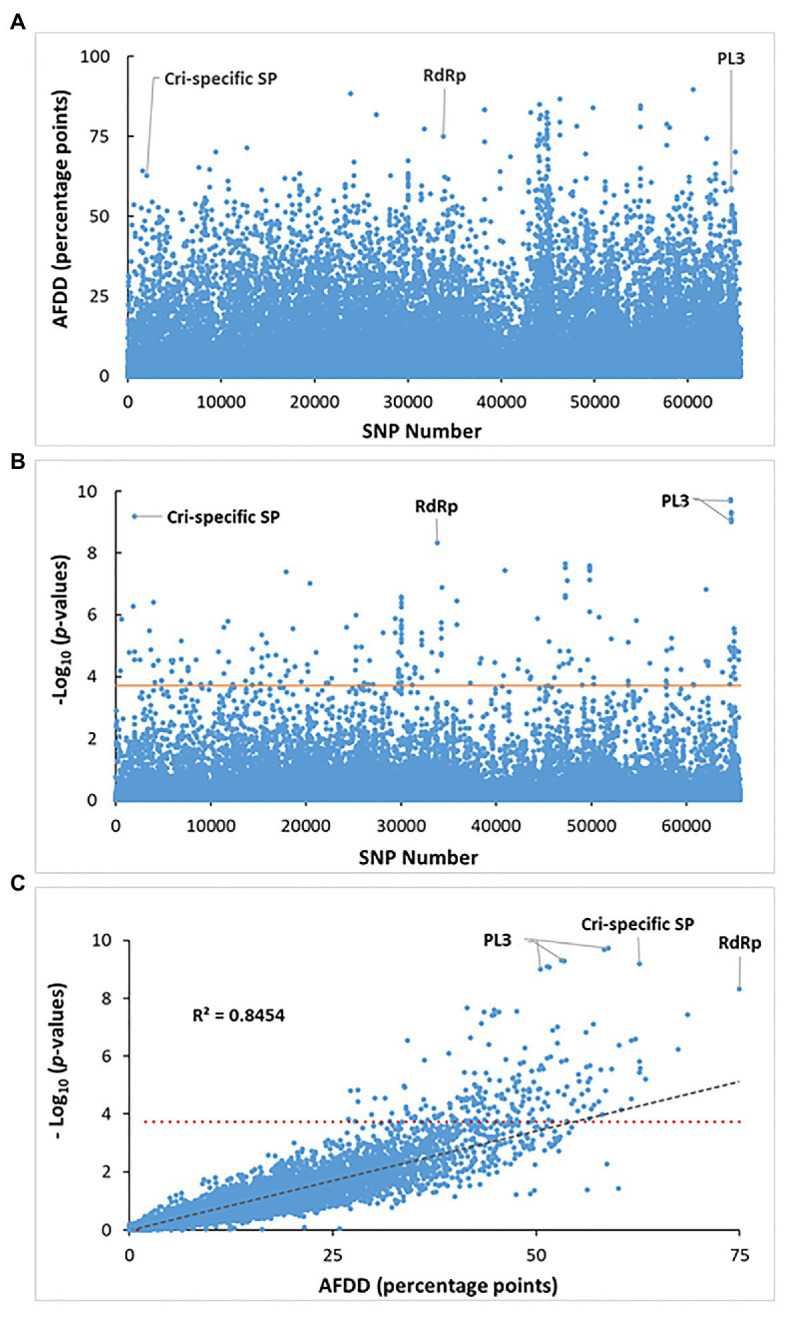
Identification of SNP loci for significant association with the virulent phenotype. **(A)** Distribution of allele frequency directional difference (AFDD) across all tested 65,617 SNPs shared between the vcr2 and wild Avcr2 pools. About 3% of total SNPs showed AFDD >30. **(B)** Manhattan plot of association *p*-values across all tested 65,617 SNPs analyzed by XP-GWAS. Three genes are labeled to show their SNPs with association at the most significant levels. **(C)** Scatter plotting AFDD values against *p* values analyzed by XP-GWAS across all tested SNPs. The horizontal red lines in **(B)** and **(C)** indicates the FDR-corrected threshold of *p* = 2e-04 for SNP loci with significant association. The gray dashed line in **(C)** shows that FADD values are highly correlated with the *p*-values analyzed by XP-GWAS for the corresponding SNP loci.

To compare genotypes between the two pathotypes, small genomic variations, including single nucleotide variant/polymorphism (SNV/SNP), multi-nucleotide variant (MNV), and insertion/deletion (InDel), were examined based on mapping reads to the *vcr2* reference transcriptome. A total of 696,284 and 609,783 DNA variants were identified for *vcr2* and *Avcr2*, respectively. The distribution of MAF showed *vcr2* had lower proportions of variants with MAF ≥ 0.3, but higher proportion of indel and ns-SNPs than *Avcr2* (*X*^2^ test, *p* < 1e-3; Supplementary Figure S3), suggesting greater genetic diversity of *Avcr2* populations obtained from sequencing data. Analysis of DNA variants, showed that both pathotypes shared 65,617 SNPs (Figure 4A), distributed within 7,749 polymorphic transcripts, averaging 4.4 SNPs/kb.

The allele frequencies for the 65,617 SNPs shared by both *vcr2* and *Avcr2* pools were calculated (Supplementary Figure S4) and used to normalize reference and alternative allele counts for XP-GWAS analysis. Of the 65,617 shared SNPs, 2011 SNPs (3% of the total) showed FADD > 30 (Figure 4A). The statistical analysis by XP-GWAS identified 204 SNP loci with significant *vcr2*-association after FDR multiple testing correction at a threshold of *p* = 2e-04 (Figure 4B). Of the 204 significantly associated SNPs, 198 of them (97% of the total) had FADD > 30 percentage points (Figures 4B,C). These associated SNPs were distributed in 119 genes (Supplementary Table S9). As shown in Figure 4C, by plotting the XP-GWAS calculated values of *p* against FADD values across all 65,617 SNPs, we demonstrated that the results were highly correlated with each other (*R*^2^ = 0.8454). Top associated SNPs were detected in three genes denoted as contig401 (1.82E-10 ≤ *p* ≥ 9.74E-10), Spr127863_c1_seq3 (*p* = 6.24E-10), and F_Contig7773 (*p* = 4.79E-9); which encoded PL3, Cri-specific SP (without any BLAST hit in the NCBI-nr database), and RNA-dependent RNA polymerase (RdRp), respectively (Figure 4; Supplementary Tables S6, S9, S10).

The 198 associated SNPs were filtered with FADD > 40 and MAF < 0.1, resulting in 87 SNPs within 52 genes (Supplementary Table S10), including 63 SNPs in 41 genes under the hypothesis of dominant avirulence and 24 SNPs in 12 genes under the hypothesis of dominant virulence. Their annotations and BLAST top-hit homologs from corresponding species are shown in Supplementary Table S10. The putative RdRp-encoding gene (F_Contig7773) was the sole one that was detected with significantly associated SNPs under both hypothesis. Of these 52 top-associated genes, four genes (SB-contig463, F_Contig7868, SB-contig401, and F_OR-Aec15165_c0_seq1) were highly polymorphic, with eight, six, and five, and five significant SNPs, respectively.

Of the 41 *vcr2*-associated genes with presumed dominant avirulence, five genes (Spr127863_c1_seq3, F_OR-Aec12603_c0_seq1, NA_HC37594_c0_seq1, SB-Spr-contig11, and SB-contig463) encoded SPs as predicted above. Notably, the most polymorphic gene SB-contig463 encoded a SP with putative reticuline oxidase activity, with the highest level of similarity (BLAST hit E-value of 4.8e-168) to a homolog from *Amborella trichopoda* (GenBank accession no. ERN08152.1). Furthermore, six of eight associated SNPs were nsSNPs (418cac/gac=His/Asp, 422atc/gtc=Ile/Val, 1156aaa/caa=Lys/Gln, 1190aac/agc=Asn/Ser, 1195tat/gat=Tyr/Asp, and 1240ccc/tcc=Pro/Ser), and may functionally alter Cri reticuline oxidase activity. Interestingly, SB-contig463 was *de novo* induced in cankered stems infected by *vcr2*, with 23-fold higher expression than in cankered stems infected by *Avcr2* (Supplementary Table S5). F_OR-Aec12603_c0_seq1 encoded an rds1p-like stress responsive protein predicted as a SP, and the BLAST top hit revealed its closest homolog (BLAST E value = 3.2e-168) in *Melampsora larici-populina* (GenBank accession no. XP_007404719.1). One of three associated SNPs was nsSNP (714ttc/ctc=Phe/Leu) in F_OR-Aec12603_c0_seq1. This gene was mainly expressed in aeciospores with very low level or no expression in the cankered stems of WWP trees. SB-Spr-contig11 was also detected with one nsSNP (481gct/act=Ala/Thr). This gene encoded a predicted SP and was annotated as a fungal-specific CFEM domain protein, with the highest similarity to a putative SP (GenBank accession no. EGG12953.1) from *M. larici-populina* but at a low homology level (BLAST E-value of 1.4e-44). The other two SP-coding genes, Spr127863_c1_seq3_m.45366 and NA_HC37594_c0_seq1, were Cri-specific without any BLAST hit when searching against NCBI nr database (Supplementary Table S10).

Of 12 *vcr2*-associated genes with presumed dominant virulence, SB-contig401 encoded a SP that was annotated as PL3 protein. Four of its five associated SNPs were ns-SNPs (56gat/ggt=Asp/Gly, 67tct/gct=Ser/Ala, 76tac/gac=Tyr/Asp, and 82gtt/att=Val/Ile). This Cri PL3 protein was highly divergent from its homologs in other fungi, with the BLAST top-hit to a *Mixia osmundae* PL3 (GenBank accession no. XP_014566663.1) at low similarity level (BLAST E-value of 2.8e-32). Its transcript was highly induced in cankered stems infected by *Avcr2* with 7-fold higher expression than that infected by *vcr2* (Supplementary Tables S6, S9, S10).

Among other top associated genes, both F_OR-Aec15165_c0_seq1 and F_Contig7868 were highly polymorphic, and they encoded a putative alpha-2 subunit of Gpa2-Guanine nucleotide-binding protein (Gpa2) and a PH domain containing protein, respectively. Both annotated functions were involved in signaling pathways. The Cri Gpa2 was a fungal conserved protein, with the highest homology (BLAST E-value of 5.4e-156) to a *Puccinia triticina* protein (GenBank accession no. OAV91176.1). Within F_OR-Aec15165_c0_seq1, three of its five-associated SNPs were nsSNPs (652tga/tgc=Stop/Cys, 691ctc/cac=Leu/His, and 1133tac/cac=Tyr/His). Only one nsSNP (1004gaa/gga=Glu/Gly) was detected in five SNPs of F_Contig7868, but its change from acidic to neutral amino acid suggests a functional variation. These putative functional variations of signaling components imply that both genes may be functional candidates contributing to *vcr2*-related virulence.

Among the top *vcr2*-associated genes (Supplementary Table S10), other annotated functions included RdRp (F_Contig7773), arginyl-tRNA synthetase (RARS, F_Contig1146), ubiquitin system component Cue (F_Contig1284), deoxyhypusine synthase (F_Contig4649), CAMK/CAMKL/KIN4 protein kinase (F_Contig7285), RraA-like protein (F_Contig811), Eisosome component PIL1-domain-containing protein (F_Contig9075), glucose-repressible alcohol dehydrogenase transcriptional effector (F_OR-Aec12969_c0_seq1), DUF21 and cystathionine beta synthase (CBS) domain containing protein (F_OR-Aec7227_c0_seq1), and splicing factor U2AF 65 kDa subunit (F_Contig4947), and others (Supplementary Table S10).

## Discussion

### An Integrative Genomic Approach for Investigating Cri Virulence

Genetics of pathogen infectivity and host resistance are of fundamental importance to pathogen persistence and patterns of disease incidence and prevalence. The present study integrated research approaches of global profiling of transcriptomes and secretomes and BSR-Seq-based association studies to elucidate molecular mechanisms and candidate effectors involved in Cri *vcr2* virulence.

Our results demonstrated that BSR-Seq was very effective for identifying *vcr2*-candidates and associated genes by comparing DNA variants between *vcr2* and *Avcr2*. Because next-generation sequencing (NGS) can detect a large number of DNA variants at an affordable price, genome-wide association study (GWAS) has been developed and widely applied to identify phenotype-associated genes for functional verification. Using GWAS, candidate effectors and other pathogenicity-related genes were identified for their potential interaction with R genes in a few crop pathosystems (Bruce et al., 2014; Plissonneau et al., 2017; Wu et al., 2017). Exploration of allele frequency through NGS-based BSA is a powerful tool for determining genetic architecture causing phenotypic changes through associated DNA markers in targeted genomic regions (Schneeberger, 2014). A key step is exploration of AFDD by comparing allele frequencies between bulked samples. AFDD mapping is widely applied to genetic mapping of genetic loci underlying both qualitative and quantitative traits (Fekih et al., 2013; Takagi et al., 2013).

Of various GWAS approaches available, we used a BSR-Seq approach to identify *vcr2*-associated genes through integration of XP-GWAS analysis and FADD mapping. By combining RNA-seq with BSA, BSR-Seq has been demonstrated as an efficient approach for fine genetic mapping of agronomic traits in crops (Trick et al., 2012; Li et al., 2013; Ramirez-Gonzalez et al., 2015; Liu et al., 2016b) and their wild relatives (Edae and Rouse, 2019). Due to enrichment of genomic variants of the expressed genes, BSR-Seq facilitates identification of the genes causing or contributing to phenotypic traits of interest. Our BSR-Seq identified over 600K DNA variants in either *vcr2* and *Avcr2* bulked samples. Of the total variants, 65K SNPs common to *vcr2* and *Avcr2* were selected to detect vcr2-linked DNA markers. Shared markers are genetically informative because elimination of false positive variants is a challenging issue in NGS data processing (Huang et al., 2015; Ribeiro et al., 2015).

Our results indicated that without availability of a species reference sequence, BSR-Seq using a *de-novo* assembled transcriptome is an advantageous option to identify genome-wide SNPs. High consistency between XP-GWAS run and FADD mapping allowed us to refine candidate vcr2 effectors in the Cri secretome. The effectors that are shared or distinct between Cri *Avcr2* and *vcr2* pathotypes will be important in understanding host resistance and WP-BR interactions. However, RNA-Seq-based SNP discovery and related BSR-Seq-based association studies have limitations, in that they probably miss SNPs with low coverage due to low transcript expression, or with locations on the non-transcribed strand (O’Brien et al., 2015). Inter-individual variation of gene expression and allele-specific gene expression also influence allele frequency estimation accuracy (Konczal et al., 2014). As Cri samples were diploid or heterokaryotic, allele specific gene expression (ASGE) could conceivably impact allele frequency estimates. Therefore, the candidate *vcr2* effectors need functional verification in a future study.

### Transcriptomic Landscapes Underlying *vcr2* Virulence

Comparative transcriptomics through enrichment analysis of GO terms revealed oxidation-reduction process, cellular response to toxic substance, and cellular oxidant detoxification as the main biological processes that are enhanced in *vcr2* pathotypes relative to *Avcr2* pathotypes, and thus may be key to *vcr2* overcoming *Cr2*-mediated MGR in WWP seedlings. Pathogenic infection is accompanied by the formation of the reactive oxygen species (ROS) in both plants and pathogens. For successful infection, fungal pathogens must activate a series of intracellular biological processes to counteract negative effects resulting from the imbalance of redox homeostasis. A large set of Cri SPs were commonly expressed in both *vcr2* and *Avcr2*, and their putative enzymatic activities included multi-copper oxidase laccase, peroxidases, L-ascorbate oxidases, SOD, and catalase, suggesting that they may act coordinately as ROS producers and scavengers for the maintenance of intracellular redox balance through a wide range of antioxidant defense mechanisms. Worth noting, several *vcr2* exclusively expressed SPs were annotated as oxidoreductase, PDOR, PDI, and other redox-associated enzymes. PDO and PDI are key players for enzymatic disulfide bond reduction, oxidation and isomerization, contributing to essential steps in the protein folding pathway and to the stability of the native state of the proteins (Ladenstein and Ren, 2006). PDI-1 assists a set of glycoproteins in folding and disulfide bonds formation during effector secretion into the apoplast of maize plants during *Ustilago maydis* infection and its deletion led to a strong reduction in virulence (Marín-Menguian et al., 2019).

In addition to ROS stresses, Cri infection also induces host cells to produce and accumulate a large array of related anti-microbial peptides and PR proteins (Liu et al., 2013), as well as defensive chemicals (Bullington et al., 2018). Therefore, pathogens have adapted a wide spectrum of gene functions to fight against plant defense responses, constituting a diverse array of pathogenicity-related components for fungi to infect a broad range of hosts (Nakajima and Akutsu, 2014). Cri SPs belonging to several functional classes, such as GHs, GTs, subtilisin-like proteases, cutinase, endo-beta-1,6-glucanase, lipase, PR-1-like, and other enzymes involved in cell wall degradation and the synthesis of secondary metabolites with potential phytotoxic effects, were shown to be expressed in a *vcr2*-specific pattern, suggesting contributions to the successful infection of WWP *Cr2*-seedlings. Many fungal SPs with activities as degradative enzymes of plant cell wall components play functional roles in pathogenicity and virulence, evidenced by their triggering of host cell-death and other plant immunity responses (Ma et al., 2015; Zhu et al., 2017a; Gui et al., 2018). Cutinases from several fungal pathogens were shown to have activity as necrotrophic effectors (Liu et al., 2016a; Wang et al., 2017; Gui et al., 2018; Lu et al., 2018). A *Fusarium oxysporum* PR-1-like protein (Fpr1) is required for full virulence of *F. oxysporum* on a mammalian host (Prados-Rosales et al., 2012). *Candida albicans* PR1-like proteins displayed sterol binding activity with a link to virulence, which may affect fungal recognition by host cells through immune evasion by masking pathogen-associated molecular patterns (PAMPs; Bantel et al., 2018). Fungal subtilisin-like proteases disrupt the physiological integrity of the hosts during penetration and colonization, and are proposed as virulence factors in pathogenic fungi (Huang et al., 2004; Bryant et al., 2009). Our profiling of Cri transcriptomes and secretomes revealed both shared and pathotype-specific transcriptional programs. Annotated molecular functions of candidate genes imply their involvement in Cri pathogenesis. SP genes identified by transcript presence/absence provide *vcr2*-candidates with a high priority for further functional dissection of the virulence mechanism in the WPBR pathosystems.

### Cri Effector Candidates and vcr2-Associated Genes

*vcr2*-associated genes were determined in either a homozygous or heterozygous status based on two separate assumptions of dominancy for either avirulence or virulence. These two assumptions are based on gene-for-gene and matching allele models, respectively, the main models representing the basic genetic mechanisms underlying plant-microbe molecular interactions (Flor, 1951; Lambrechts et al., 2006; Thrall et al., 2016). Gene-for-gene interaction assume that vcr2 overcomes MGR through escaping recognition by the WWP *Cr2* gene. The WWP *Cr2* protein is probably an immune receptor encoded by a NBS-LRR gene that specifically interacts with the corresponding *Avcr2* effector expressed by Cri (Liu et al., 2017). Interaction of an avirulent (Avr) effector and its specific R protein resulting in incompatibility is known as effector-triggered immunity (Jones et al., 2016). Therefore, evolution of a rust virulent race (such as *vcr2*) is thought to involve a mutation (including deletion) of an Avr gene for a loss of function. Absence of the Avr effector leads to the pathogen being unrecognizable by the corresponding host R protein, resulting in successful infection of previously resistant hosts (Dodds and Rathjen, 2010).

Annotation of significantly associated SNPs revealed six SPs as effector candidates: reticuline oxidase, rds1p-like stress response protein, fungal-specific CFEM domain protein, PL3, and two Cri-specific SPs (Spr127863_c1_seq3 and NA_HC37594_c0_seq1) without any annotated function.

Reticuline oxidase belongs to the super-family of the berberine bridge enzymes (BBEs) found in bacteria, fungi, and plants. Six of eight associated SNPs in the Cri BBE homolog were ns-SNPs (the highest FADD = 51 with FDR-corrected *p* = 0.003). In addition, *in planta* expression of the Cri BBE homolog was 23-fold higher in *vcr2* than in *Avcr2*. Nonsynonymous mutations of *Avr* genes have resulted in an acquired virulence for several R genes (Mesarich et al., 2014; Chen et al., 2017; Salcedo et al., 2017). BBEs are flavoenzymes that catalyze carbohydrate oxidation, either for the biosynthesis of berberine type alkaloids, or for H_2_O_2_ generation (Daniel et al., 2017). Several fungal BBEs showed activity for oxidation of oligosaccharides degraded from chitin and xylan. As oligosaccharides are fungal elicitors, their deactivation would lead to downregulation of the plant immune response (Daniel et al., 2017). Among fungal BBEs with oxidation activity, a *Phytophthora infestans* BBE-like enzyme (PITG_02930) proved to be required for the invasion of host plants (Raffaele et al., 2010). On the other hand, a few fungal BBEs catalyze complex reactions for biosynthesis of alkaloids that are involved in anti-biotic mechanisms (Daniel et al., 2017).

Fungal-specific CFEM domain proteins contain eight cysteines. They were highly divergent across different species and have been proposed to play roles in fungal pathogenesis (Kulkarni et al., 2003). A recent study reported contribution of a *Botrytis cinerea* CFEM domain protein (BcCFEM1) to virulence, conidial production, and stress tolerance (Zhu et al., 2017b). The Cri rds1p-like protein contained a ferritin-like domain, belonging to the Ferritin-like superfamily, whose role in defense against toxic oxygen species are well documented (Andrews, 2010). The Cri rds1p-like gene was highly expressed in aeciospores and urediniospores, but at a low to no expression levels in cankered pine stems, suggesting that it may play a role during interactions of Cri with its alternative host plants.

PL3 enzymes hydrolyze long-chain acyl-triglycerides into di‐ and monoglycerides, glycerol, and free fatty acids, and their activities appear to be important for the pathogenicity of several fungi (Gaillardin, 2010). A Cri PL3 homolog was identified with two ns-SNPs under the assumption of virulence dominancy. This gene was also among DEG, with an expression level 7-fold higher in *Avcr2* than in *vcr2*, suggesting its potential role as effector in avirulence. Several fungal PLs have been implicated as having effector roles in plant pathogens (Ben-Daniel et al., 2011; Yang et al., 2018). Additionally, almost all effectors are highly expressed *in planta* to play important roles in pathogenicity and virulence (Mesarich et al., 2014). *In planta* upregulation of effector expression may help fungal mycelia to extract nutrition, in addition to the more obvious role of physically facilitating invasion of the host tissue. Consistently, both Cri BBE and PL3 homologs are highly expressed in cankered stems relative to their expression during the aeciospore stage.

In addition to SPs, annotations of other top *vcr2*-associated genes implied their involvement in virulence, including PH domain containing protein, Gpa2, RdRp, arginyl-tRNA synthetase, deoxyhypusine synthase, eisosome component PIL1-domain-containing protein, and DUF21 and CBS domain containing protein. PH domain proteins play a role in intracellular signaling or as constituents of the cytoskeleton (Lemmon and Ferguson, 2000). The PH domain has been found in different proteins, such as regulators of small G-proteins and G protein receptor kinases (Fort and Blangy, 2017; Komolov and Benovic, 2018). A *Cryptococcus neoformans* PH domain containing protein (Cin1) was found to have pleiotropic functions in morphogenesis, endocytosis, exocytosis, and fungal virulence (Wang and Shen, 2011). Gpa2 is another modulator with roles in various transmembrane signaling systems, including response to environmental nutrients (Harashima et al., 2006), regulation of fungal morphogenesis and hypha formation (Miwa et al., 2004), and heat resistance (Kraakman et al., 1999).

Mutations of aminoacyl-tRNA synthetase (ArgRS) genes have been implicated in a spectrum of inherited, single-gene (Mendelian) human disorders (Oprescu et al., 2017). One of Cri ARS (Ile-tRNA synthetase gene) was specially expressed in *vcr2*-infected cankered stems. Arginyl-tRNA synthetase is well known because its homozygous variant causes a rare Mendelian recessive disorder called Pelizaeus-Merzbacher disease in humans (Nafisinia et al., 2017). Overexpression of plant proteins containing CBS and DUF21 domains conferred higher tolerance against various abiotic stresses such as salinity, heavy metals, oxidative stress, and low nitrogen conditions (Singh et al., 2012; Hao et al., 2016). Deoxyhypusine synthase catalyzes the biosynthesis of hypusine, a modification of a specific lysine residue in the precursor of eukaryotic translation initiation factor 5A (eIF-5). A yeast deoxyhypusine synthase mutant (*dys1-1*) exhibited defects associated with protein synthesis, translation machinery, and cell wall integrity; and a complete growth dependency on an osmotic stabilizer (1 M sorbitol; Galvao et al., 2013). Fungal proteins Pil1 and Lsp1 are key components for formation of eisosomes. At least another 17 proteins including two PH domain containing proteins, were detected in the eisosomes. Eisosome mutants display abnormal spatial regulation of cell wall synthesis, higher sensitivity to a variety of oxidants, and a greatly reduced virulence (Foderaro et al., 2017). RdRps are key components of RNA viruses. Several RNA mycoviruses are well documented for the harmful effect on their host fungi by causing mitigation of fungal virulence or hypovirulence (Hillman et al., 2018). A previous study showed a Cri totivirus (CrTV4) with significant association with *vcr2* virulence (Liu et al., 2019), supporting a proposed hypothesis of cytoplasmic inheritance of Cri virulence (Kinloch et al., 2004). All of the *vcr2*-associated functions might target genomic regions which are potentially responsible for Cri virulence adaptation. Additional annotations of the top *vcr2*-associated genes were CAMK/CAMKL/KIN4 protein kinase, RraA-like protein, glucose-repressible alcohol dehydrogenase transcriptional effector, and splicing factor U2AF 65 kDa subunit. However, networking of these functions with pathogenic virulence awaits future studies.

Currently identification of Cri virulence pathotypes is time-consuming, totally dependent on a race’s ability to overcome resistance of MGR trees with well-known genotypes by natural infection or controlled inoculation. Through this approach, we collected Cri samples of the *vcr2* pathotype from both western and eastern Oregon, several areas of the latter region were previously surveyed but neither *vcr2* nor *Cr2* was found (Kinloch et al. 2003, 2004). Our inoculation utilized very high spore loads (>6,000 spores/cm^2^) on MGR seedlings, allowing visualization and sampling of *vcr2* races even though they were presumably at low frequency in a heterogeneous mixture from eastern Oregon. The identification of effector candidates and *vcr2*-associated genes would allow future efforts to isolate the virulent effectors and determine their functions for development of diagnostic tools for monitoring Cri virulence across North America. The SNP assay will be developed from *vcr2*-associated genes, which could be useful in the future for monitoring for presence of *vcr2*, or determining if *vcr2* is present in infected stems of *Cr2*/– genotypes rather than potential other explanations. Discovery of a large number of genes as well as related biological processes contributing to Cri pathogenicity provides a valuable insight into the comprehensive understanding of molecular WP-BR interactions.

## Data Availability Statement

All sequencing data generated in this study are available from the SRA-Archive (http://www.ncbi.nlm.nih.gov/sra) under the study accession SRP031625. Raw reads of the Illumina RNA-seq 100-bp PE sequences from nine samples were deposited in the NCBI SRA under accession number SRR11101722‐ SRR11101730. A *Cronartium ribicola* vcr2 reference transcriptome was deposited at DDBJ/EMBL/GenBank under TSA accession GIKE00000000. All other supporting data are included in the Supplementary Material.

## Author Contributions

J-JL and RS conceived the research and supervised the project. RS, AK, and DS provided the genetic materials, conducted the disease ratings, and analyzed phenotypic data. J-JL, AZ, HW, and KO conducted biochemical experiments and analyzed NGS data. J-JL wrote the original draft with contributions from all authors. All authors contributed to the article and approved the submitted version.

### Conflict of Interest

The authors declare that the research was conducted in the absence of any commercial or financial relationships that could be construed as a potential conflict of interest.

## Footnotes

^1^http://www.cbs.dtu.dk/services/TMHMM/

^2^https://www.ebi.ac.uk/Tools/hmmer

^3^https://cran.r-project.org/web/packages/gap/index.html

## Supplementary Material

Supplementary Table S1 Statistics of RNA-seq reads and gene expression analysis of reads mapped back to the *vcr2* reference transcriptome.

Supplementary Table S2 Statistics of the *vcr2* reference transcriptome generated in the present study.

Supplementary Table S3 Output of OrthoVenn2 ortholog cluster analysis of *Cronartium ribicola vcr2* and *Avcr2* pathotypes.

Supplementary Table S4 GO-term enrichment analysis of pathotype-unique clusters detected by OrthoVenn2.

Supplementary Table S5 Amino acid sequences of 1014 secreted proteins predicted in *Cronartium ribicola* transcriptomes.

Supplementary Table S6 Annotation and expression levels of 1014 proteins identified in the *Cronartium ribicola* secretome.

Supplementary Table S7 Enriched GO-terms of pathotype-specifically expressed genes and differentially expressed genes as detected by RNA-seq Analysis.

Supplementary Table S8 *Cronartium ribicola* unigenes with the most abundant transcripts (FPKM > 1,000) expressed in cankered stems of western white pine with the *Cr2*/− genotype.

Supplementary Table S9 Putative protein sequences of 119 associated genes.

Supplementary Table S10 Annotation of 87 top *vcr2*-associated SNPs in 52 polymorphic genes.

Supplementary Figure S1 Distributions of transcripts and RNA-seq reads for the transcriptomes *de-novo* assembled from *Cronartium ribicola vcr2* samples. Western white pine transcripts from *vcr2*-infected host tissues were pre-removed by BLASTn search against transcriptomes of healthy host tissues. MEGA analysis assigned all transcripts into four categories (fungi, no hits, not assigned, and other). **(A)** Distribution of transcripts across four categories; **(B)** Distribution of the mapped RNA-seq reads across four categories.

Supplementary Figure S2 Ortholog clusters analysis between *Cronartium ribicola vcr2* and *Avcr2* pathotypes using OrthoVenn2 (Xu et al., 2019).

Supplementary Figure S3 Comparisons of minor allele frequency (MAF) distributions between *vcr2* and *Avcr2*. **(A)** Distribution of minor allele frequency (MAF) of all DNA variants. A total of 696K and 609K DNA variants with site coverage >10 were analyzed in *vcr2* and *Avcr2*, respectively. **(B)** MAFs of 65,617 SNPs shared between *vcr2* and *Avcr2*. **(C)** Three types of DNA variants (SNV/SNP, MNV, and Indel). **(D)** Two types of SNPs (synonymous-SNP and nonsynonymous-SNP) detected in the coding regions. Three stars indicate significant difference between *vcr2* and *Avcr2* (*X*^2^ test at *p* < 0.001).

Supplementary Figure S4 Distribution of allele frequency (AF) of 65,617 SNPs shared by *vcr2* and *Avcr2*.
