# Supplementary figures and images for: Comparative Transcriptomics and RNA-Seq-Based Bulked Segregant Analysis Reveals Genomic Basis Underlying Cronartium ribicola vcr2 Virulence
Source: Front Microbiol. 2021 Feb 22;12:602812. doi: 10.3389/fmicb.2021.602812 (PMC7990074; doi:10.3389/fmicb.2021.602812)

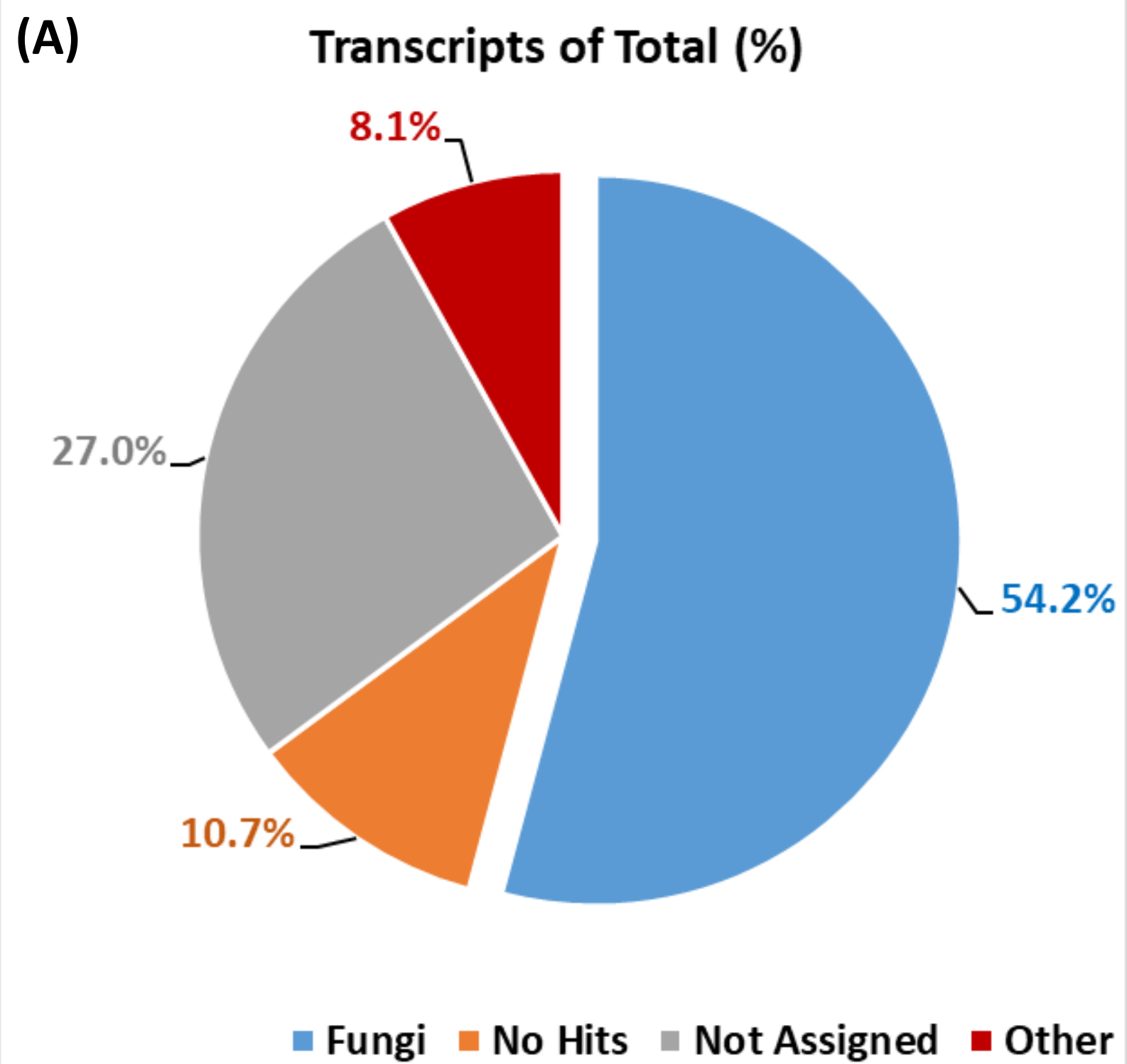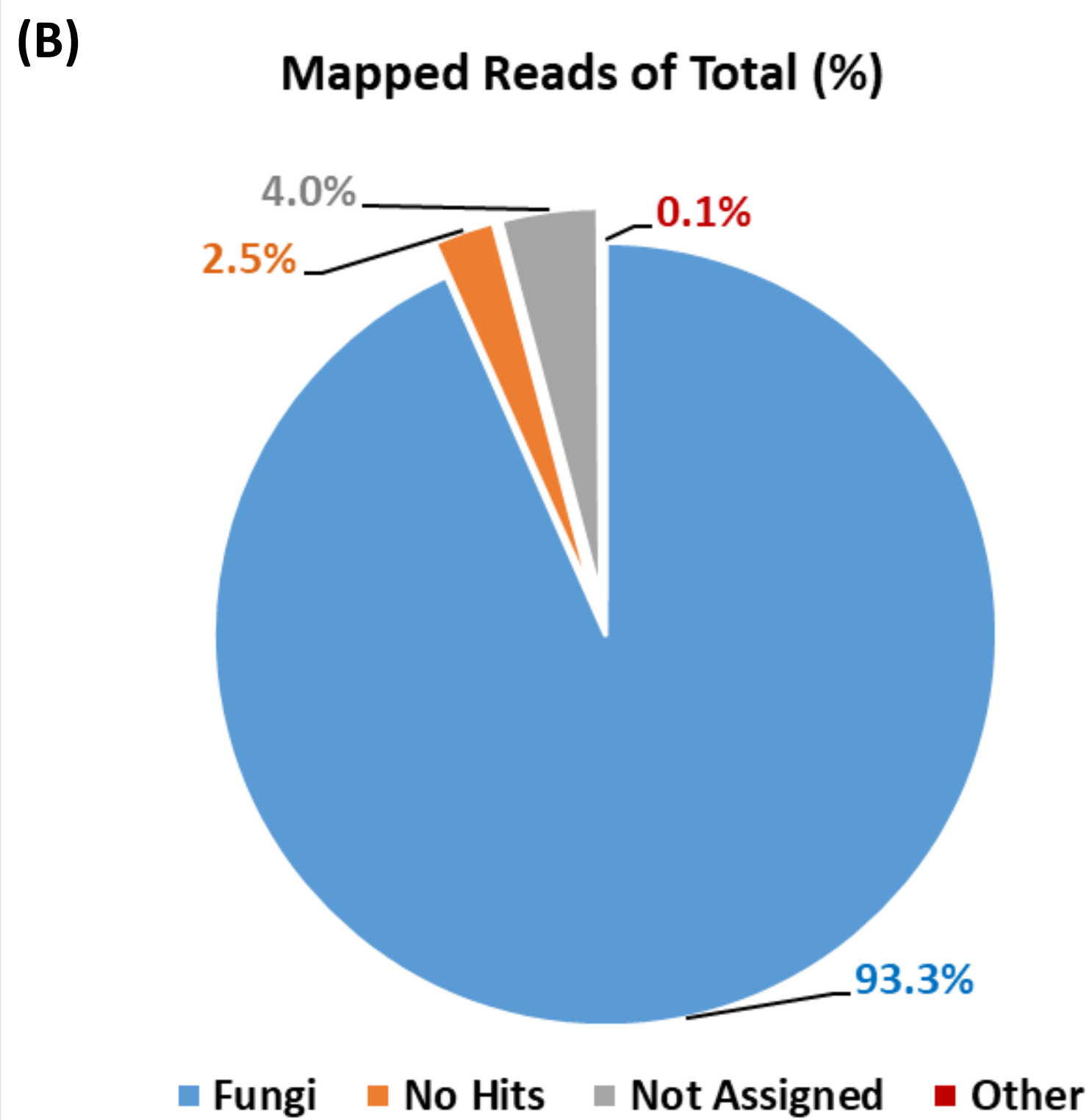

Figure 1

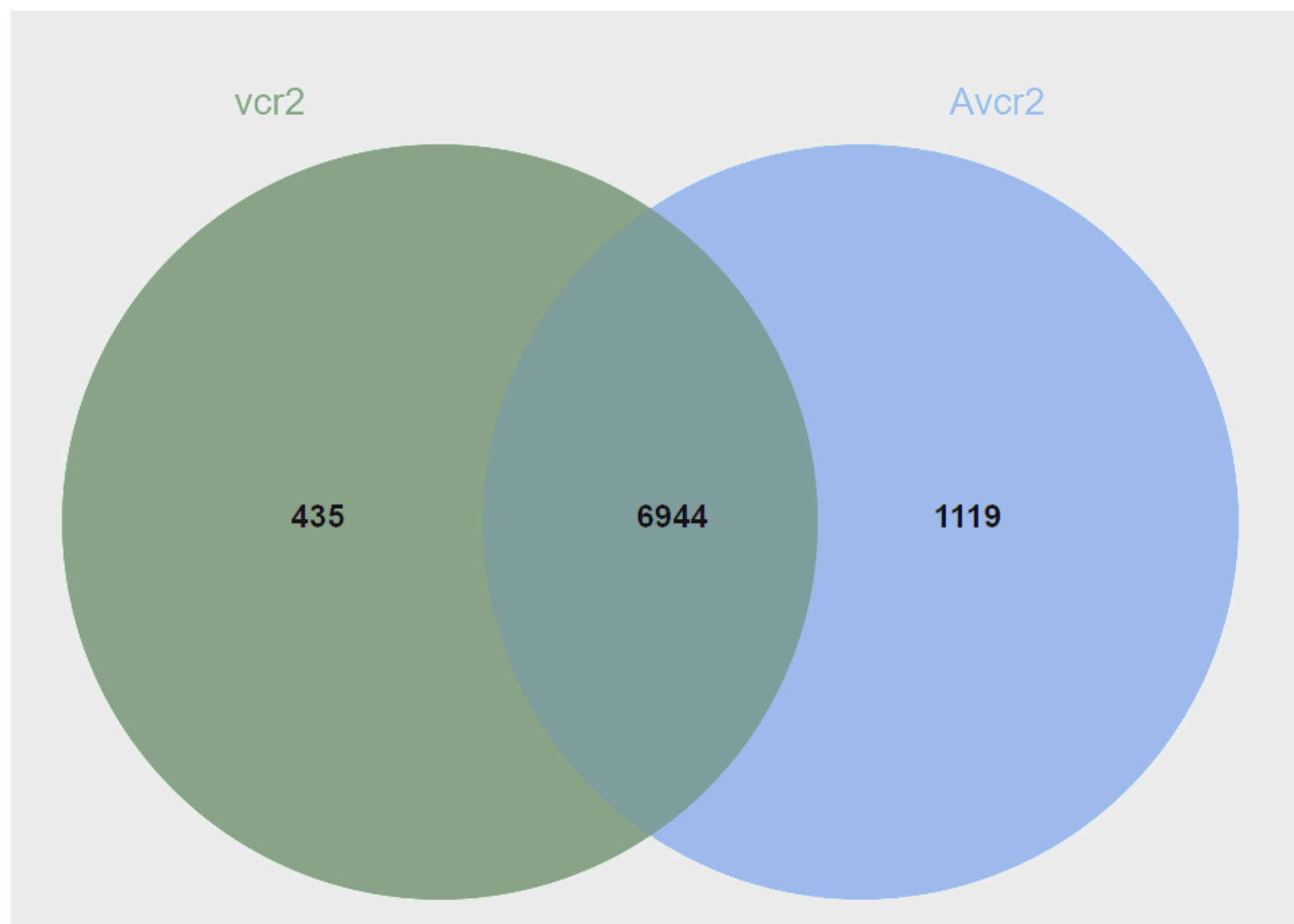

Figure S2



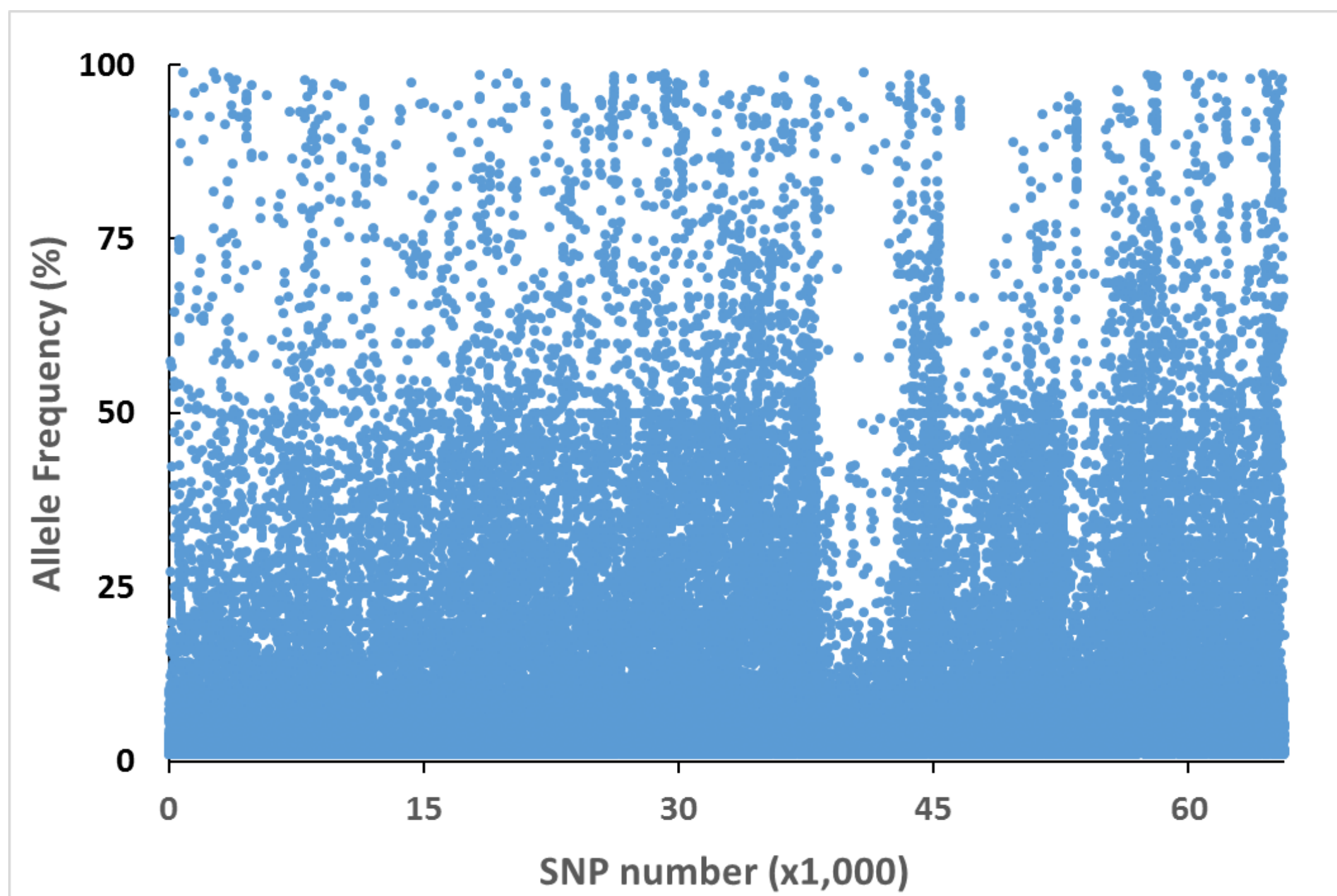

Figure S4

Supplement: Supplementary Figure S1 — Distributions of transcripts and RNA-seq reads for the transcriptomes de-novo assembled from Cronartium ribicola vcr2 samples. Western white pine transcripts from vcr2-infected host tissues were pre-removed by BLASTn search against transcriptomes of healthy host tissues. MEGA analysis assigned all transcripts into four categories (fungi, no hits, not assigned, and other). (A) Distribution of transcripts across four categories; (B) Distribution of the mapped RNA-seq reads across four categories. [file Data_Sheet_1.pdf]
